# Factors associated with the timely uptake of initial HIV virologic test among HIV-exposed infants attending clinics within a faith-based HIV program in Kenya; a cross-sectional study

**DOI:** 10.1186/s12889-021-10587-1

**Published:** 2021-03-23

**Authors:** Douglas Gaitho, Freda Kinoti, Lawrence Mwaniki, Diana Kemunto, Victor Ogoti, Catherine Njigua, Elizabeth Kubo, Agnes Langat, Jared Mecha

**Affiliations:** 1 Christian Health Association of Kenya (CHAK), P.O. Box 30690 – 00100, GPO, Nairobi, Kenya; 2 Department of Clinical Medicine & Therapeutics, University of Nairobi, P.O. Box 19676 – 00202, Nairobi, Kenya; 3 Division of Global HIV and TB, Centers for Disease Control and Prevention Kenya, P.O. Box 606 – 00621, Village Market, Nairobi, Kenya; 4 Department of Clinical medicine & Therapeutics, College of Health Sciences – University of Nairobi, P.O. Box 19676 – 00202, Nairobi, Kenya

**Keywords:** HIV-exposed infant, Early infant diagnosis

## Abstract

**Background:**

Early infant diagnosis (EID) of HIV, followed by effective care including antiretroviral therapy (ART), reduces infant mortality by 76% and HIV progression by 75%. In 2015, 50% of 1.2 million HIV-exposed infants (HEI) in 21 priority countries received a virologic test within the recommended 2 months of birth. We sought to identify factors associated with timely uptake of virologic EID among HEI and gain insight into missed opportunities.

**Methods:**

This was a cross-sectional study that used de-identified data from electronic medical records of 54 health facilities within the Christian Health Association of Kenya (CHAK) HIV Project database. All HEI who had their first HIV virologic test done between January 2015 and December 2017 were included in the study and categorized as either having the test within or after 8 weeks of birth. Multivariate linear mixed effects regression model was used to determine factors associated with uptake of the first HIV EID polymerase chain reaction (PCR). Predictor variables studied include sex, birth weight, the entry point into care, provision of ART prophylaxis for the infant, maternal ART at time of EID, mode of delivery, and place of delivery.

**Results:**

We included 2020 HEI of whom 1018 (50.4%) were female. A majority, 1596 (79.0%) had their first HIV PCR within 2 months of birth at a median age of 6.4 weeks (interquartile range 6–7.4). Overall, HIV positivity rate at initial test among this cohort was 1.2%. Delayed HIV PCR testing for EID was more likely to yield a positive result [adjusted odds ratio (aOR) = 1.29 (95% confidence interval (CI) 1.09–1.52) *p* = 0.003]. Infants of mothers not on ART at the time of HIV PCR test and infants who had not received prophylaxis to prevent vertical HIV transmission had significant increased odds of a delayed initial test [aOR = 1.27 (95% CI = 1.18–1.37) *p* = < 0.0001] and [aOR = 1.43 (95% CI 1.27–1.61) *p* = < 0.001] respectively.

**Conclusion:**

An initial HIV PCR test done after 8 weeks of birth is likely to yield a positive result. Barriers to accessing ART for treatment among HIV-infected pregnant and breastfeeding women, and prophylaxis for the HEI were associated with delayed EID. In order to ensure timely EID, programs need to incorporate both facility and community strategy interventions to ensure all pregnant women seek antenatal care and deliver within health facilities.

## Background

Early infant diagnosis (EID) is the critical first step in reducing human immunodeficiency virus (HIV)-related infant mortality through prompt identification of HIV-infected infants and subsequent initiation of antiretroviral therapy (ART) [1, 2]. Data from the Children with HIV Early Antiretroviral Therapy (CHER) trial showed that early diagnosis of HIV reduced infant mortality by 76% and HIV progression by 75% [3]. Mother-to-child transmission (MTCT) of HIV accounts for the majority (> 95%) of infections among children with a 30% transmission rate if no prevention interventions are offered [4–7]. Missed opportunities for EID among those infected during pregnancy or labor and delivery results in the death of a third of the HIV-infected infants by the age of 1 year and 50% by 2 years due to severe malnutrition and opportunistic infections [8, 9]. Describing factors associated with uptake of virologic testing for EID of HIV is vital for designing strategies to prevent missed opportunities.

In sub-Saharan Africa, MTCT of HIV remains unacceptably high, though significant declines have been recorded in 21 priority countries that account for 90% of the global HIV infections among HIV-infected pregnant women [10]. In these priority countries, MTCT rates declined from 22.4 to 8.9% between 2009 and 2015 [11]. However, in Kenya, MTCT rates increased from 8.3% in 2015 to 11.5% in 2017 among an estimated 69,497 HIV-infected pregnant women [11–13].

The World Health Organization recommends the use of a polymerase chain reaction (PCR) assay as the preferred test for EID within 4–6 weeks of birth for HIV-exposed infants (HEI) [14–16]. The Kenya national guidelines have adopted this recommendation, with the preferred timing for EID at 6 weeks, coinciding with the first routine immunization visit [17]. EID testing at 6 weeks is cost effective and contributes to dramatic improvements in mortality and morbidity in HIV-infected infants and children [18]. However, in 2015, only 50% of 1.2 million HEI within the priority countries received a virologic test within the recommended time frame [11]. This scenario is similar in Kenya where according to 2017 estimates, only 51% of HEI had EID within 2 months of birth [19].

Barriers to uptake of EID include lack of integration of prevention of MTCT (PMTCT) services within Maternal and Child Health (MCH) clinics, supply chain management, referral and networking of specimens, blood collection to results turnaround time and inability to reach children who are not in the healthcare system due to loss to follow up of mothers and infants between the time of HIV diagnosis of the mother and post-partum return for EID [20–26]. Addressing health system barriers, defining a comprehensive package for HEI, and the provision of integrated services are essential to the success of the EID program [27]. Meaningful impact of these programs on child health outcomes requires innovative interventions that take into consideration different contexts, epidemiology, and health systems [28, 29]. There is a paucity of data on factors associated with delayed virologic testing for EID within programmatic settings in Kenya. We sought to identify factors associated with uptake of the initial HIV PCR test for EID of HIV, in order to develop data-driven programmatic initiatives to eliminate missed opportunities.

## Methods

### Study design

This was a cross-sectional study that used de-identified data from electronic medical records (EMR) of 54 faith-based health facilities within the Christian Health Association of Kenya (CHAK) HIV Project.

### Study setting

The study was conducted in 54, mostly rural, faith-based health facilities in Kenya. Of these, 44 facilities use point-of-care EMR, whereas ten use a hybrid system whereby patient data are transferred from paper-based charts into the EMR system. The National program has implemented option B+ for HIV pregnant and breastfeeding mothers since 2013 in line with the Kenya national guidelines [30]. All pregnant and breastfeeding women have their HIV status established at first clinical contact. For women newly identified as HIV positive, ART is initiated preferably on the same day or within 2 weeks of diagnosis and infant ART prophylaxis is issued. An EID virologic test is offered for all HEI at the 6-week immunization visit or at first contact thereafter. Infants who present with signs and symptoms suggestive of HIV infection, however, may receive EID testing earlier. All facilities collect dried blood spot samples for HIV PCR and are sent to the nearest reference laboratory.

### Study population

We enrolled all HEI who had a dried blood spot sample collected from a fingerprick between January 2015 and December 2017. We excluded repeat or confirmatory tests and those missing EID testing outcomes or predictor data.

### Study variables

The primary study outcome variable was a binary response- of either early or delayed initial HIV PCR testing. A test was categorized as early if the sample was collected before or at 8 weeks of birth. In accordance with national and programmatic evaluations, an HIV PCR done after 8 weeks was considered a late initial test [11].

Predictor variables studied include infant sex, birth weight (low birth weight was defined as a weight below 2500 g), entry point into care (the on-site HIV Clinic, MCH clinic, maternity, emergency out-patient department or pediatric clinic), provision of ART prophylaxis for the infant (yes or no), maternal ART at time of EID (regardless of whether this was initiated before pregnancy, during pregnancy, or after delivery prior to EID), mode of delivery (cesarean or vaginal), and place of delivery (skilled or unskilled).

### Data collection and analysis

The study data were extracted from a de-identified database of EMR of 54 health facilities. Descriptive statistics were computed for the study population. Continuous variables (time to HIV PCR and age at enrollment of the infant), and frequencies and proportions for categorical variables (sex, entry point into care, mode of delivery, place of delivery, maternal ART, infant ART prophylaxis and birth weight) were calculated. To determine factors associated with a late initial HIV PCR uptake, bivariate analysis and mixed-effect multivariable linear regression were conducted using STATA. Crude and adjusted odds ratio (aOR), with corresponding 95% confidence intervals (CIs) are presented. All *p* values were based on a two-tailed test, with a 5% level of significance. Analysis took into consideration missing values and intra-cluster correlation. To handle missing data, multivariate imputation methods were utilized to impute all clusters simultaneously. We fitted a linear mixed effects model and ran the Odds ratio estimate command.

## Results

We included 2020 HEI in the study where half (50.4%) of the infants were female with a median age of 1 month at enrollment (interquartile range (IQR) 1–2). Of the HEI, 1596 (79.0%) had their first HIV PCR within the recommended 8 weeks of birth. Median time to initial virologic test was 6.4 weeks (IQR 6–7.4). Most infants were delivered in health facilities (73.4%); vaginal deliveries were 1267 (62.7%). Entry point into care for over half of the infants was the MCH clinic (53.1%). Uptake of maternal ART and provision of infant prophylaxis remained sub-optimal with 5.8 and 3.3% not having received this intervention at the time of initial HIV PCR respectively. The positivity rate at first HIV PCR was 1.2% (Table 1).

**Table 1:** Characteristics of the HIV exposed infants

Characteristic	Frequency/median	Percentage (%)
Sex		
Male	1002	49.6
Female	1018	50.4
Birth weight		
Underweight (< 2500 g)	184	9.1
Normal weight	1761	87.2
Missing (undocumented)	75	3.7
Age at enrollment in care (median IQR)	6 weeks	[5–7] weeks
Source of referral		
CCC	683	33.8
MCH/PMTCT	1073	53.1
Maternity	71	3.5
OPD	20	1.0
Pediatric clinic	6	0.3
Other	33	1.6
Unknown	134	6.6
ARV Prophylaxis for infant		
Yes	1609	79.7
No	66	3.3
Missing	345	17.1
Mother on ART		
Yes	1459	72.2
No	118	5.8
Missing	443	21.9
Mode of delivery		
Vaginal	1267	62.7
C-section	391	19.4
Missing	362	17.9
Place of delivery		
Facility	1483	73.4
Home	116	5.7
Missing	421	20.8
First PCR result		
Negative	1557	77.1
Positive	19	0.9
Missing	444	22.0

A delayed HIV PCR for EID was more likely to yield a positive result [aOR = 1.29 (95% CI 1.09–1.52) *p* = 0.003]. Mothers not on ART at the time of HIV PCR test had significantly increased odds of a delayed HIV PCR test [aOR = 1.27 (95% CI 1.18–1.37) *p* < 0.0001], whereas infants who did not receive prophylaxis had almost twice the likelihood of having late testing after 8 weeks of birth [aOR = 1.43 (95% CI 1.27–1.61) *p* < 0.001]. Home delivery was associated with a delayed first HIV PCR on univariate analysis [OR = 1.16 (95% CI 1.07–1.26) *p* = 0.001] however this was not significant in the multivariate model [aOR = 1.07 (95% CI 0.99–1.17) *p* = 0.089] (Table 2).

**Table 2:** Factors associated with a delayed initial HIV PCR test

Variable	PCR test (weeks)		Crude OR (95%CI)	*P* value	AOR (95% CI)	*P* value
	PCR in 8 weeks	PCR after 8 weeks				
Sex						
Male	799	203	Ref		Ref	
Female	797	221	1.01 (0.98–1.05)	0.42	1.01 (0.98–1.05)	0.35
Birth weight						
2500+ g	1412	349	Ref		Ref	
< 2500 g	135	49	1.06 (0.99–1.13)	0.07	1.03 (0.97–1.09)	0.25
ARV prophylaxis (infant)						
Yes	1322	287	Ref		Ref	
No	21	45	1.68 (1.53–1.86)	**< 0.0001**	1.43 (1.27–1.61)	**< 0.0001**
Mother on ART						
Yes	1225	234	Ref			
No	58	60	1.44 (1.34–1.55)	**< 0.0001**	1.27 (1.18–1.37)	**< 0.0001**
Mode of delivery						
Vaginal	1022	245	Ref		Ref	
C-section	328	63	0.98 (0.94–1.03)	0.50	1.02 (0.98–1.07)	0.325
Place of delivery						
Facility	1221	262	Ref		Ref	
Home	77	39	1.16 (1.07–1.26)	**0.001**	1.07 (0.99–1.17)	0.089
1st PCR result						
Negative	1226	331	Ref		Ref	
Positive	7	12	1.47 (1.23–1.76)	**< 0.0001**	1.29 (1.09–1.52)	**0.003**

## Discussion

The uptake of initial HIV PCR testing within our study population was 79.0%, which was higher than the national average of 51.0% per 2017 estimates [12]. The program has deliberately implemented interventions to support EID testing and efficiency such as sample networking, the use of mentor mothers and community health volunteers to ensure pregnant and breastfeeding women are followed up at the facility through referrals and defaulter tracing, capacity building initiatives, mentorship and on-job training all to ensure a robust and efficient EID program. These strategies have contributed to the median age at HEI PCR of 6.4 weeks which is similar to 6.7 weeks reported in Ethiopia [31]. In Uganda, a mentor mother program showed increased retention in care with overall reduction in incidence of lost to follow-up throughout the PMTCT cascade with subsequent good EID uptake [32].

Despite this, EID uptake within the first 2 months of birth in the program remains suboptimal. Challenges associated with delayed initial HIV PCR include pregnant and breastfeeding women and infants not on ART for treatment and prophylaxis respectively at the time of HIV PCR testing or home delivery. All healthcare facilities providing PMTCT services ought to be networked to reference laboratories that conduct PCR testing to ensure short turnaround times between sample collection and delivery of results [33]. Provision of infant prophylaxis is cornerstone in preventing HIV transmission in the postnatal period [34].

Lack of maternal ART at the time of EID was significantly associated with late EID testing. These findings are similar to those of a retrospective cohort study in India where the odds of a delayed HIV PCR was double among HIV breastfeeding women not on ART [35]. Published literature has shown how missed opportunities for HIV testing of pregnant women in antenatal clinics, labor, and delivery significantly contribute to HIV pregnant women not initiating ART [36, 37]. Integrating PMTCT services within existing maternal and child health services is key to a successful program [38]. To address the critically linked issues of maternal HIV testing, ART, HEI EID testing, programs ought to develop strategies to address health system gaps in service accessibility, and quality, staffing required for service delivery, and community-level factors that include addressing stigma, patient literacy, and HIV status disclosure to partners and their support [39, 40]. A national survey conducted in Kenya in 2017, demonstrated how non-disclosure of HIV status led to increased HIV transmission among infants [41]. Programs taking care of HIV pregnant or breastfeeding women need to facilitate HIV status disclosure to partners and encourage male involvement if elimination of mother to child transmission is to be achieved [42]. Community support to ensure exclusive breast feeding of infant have been shown to significantly reduce MTCT of HIV [43].

Home delivery was associated with a delayed uptake of EID testing on univariate analysis. According to the 2014 Kenya Demographic and Health Survey, 37% of births occur at home [44]. This proportion is higher than what we observed from our participants. Home delivery is a missed opportunity to diagnose maternal HIV infection and infant HIV exposure status. A retrospective cohort study conducted in Ethiopia demonstrated that half of infants delivered at home did not receive prophylaxis and was associated with a delay in initial HIV PCR [45]. Increasing facility-based delivery is paramount for an efficient and effective EID program [46]. Socioeconomic factors have been shown to play a major role in facilitating access to maternal and child health services. Women from a low socioeconomic background are more likely to have fewer antenatal clinic visits and have unskilled birth attendants [47]. Advocacy towards intensified political and financial commitment towards PMTCT programs is essential in protecting lives of children – it’s effective and improves access to services while averting unnecessary morbidity and mortality [48].

### Limitations

Given the nature of the study design, we were not able to control for certain potential confounding factors as data on these had not been collected. These factors include but are not limited to socioeconomic status, mother’s level of education, distance from the health facility, marital status, missed opportunities within health facilities and turnaround time of results. Infant feeding practices have also been shown to play a significant role in HIV transmission among infants [49]. We did not include confirmatory test results for samples that had an initial positive outcome. Given the retrospective study, missing data was an identified gap, however statistical techniques to avoid the introduction of bias were employed.

## Conclusion

Our program had better uptake of EID (79.0%) as compared with the national program (51%). Barriers to accessing ART for treatment among HIV-infected pregnant and breastfeeding women, prophylaxis for the HEI and home delivery were associated with delayed EID. In order to ensure timely EID, programs need to incorporate both facility and community strategy interventions to ensure all pregnant women seek antenatal care and deliver within health facilities. These interventions will help ensure HIV-positive pregnant and breastfeeding women are identified, initiated on ART, their infants provided with prophylaxis, and their HEI receive timely EID testing.

## Data Availability

Public access to the database is closed. However, the deidentified data set used and/or analyzed are available from the corresponding author on reasonable request.

## Abbreviations

ART: Antiretroviral therapy
aOR: Adjusted odd ratio
CI: Confidence interval
CHAK: Christian Health Association of Kenya
EID: Early infant diagnosis
EMR: Electronic medical records
HEI: HIV-exposed infants
HIV: Human immunodeficiency virus
IQR: Interquartile range
MCH: Maternal and Child Health
MTCT: Mother-to-child transmission
PEPFAR: President’s Emergency Plan for AIDS Relief
PMTCT: Prevention of mother-to-child transmission
